# Supplementary material for: Polyketide synthase gene clusters in the ericoid mycorrhizal fungus Oidiodendron maius: genome-wide prediction, expression profiling, and functional characterization of knockout mutants
Source: Front Plant Sci. 2026 Sep 16;17:1924430. doi: 10.3389/fpls.2026.1924430 (PMC13623903; doi:10.3389/fpls.2026.1924430)
Supplement: Supplementary file 1 [file SupplementaryFile1.zip › Supplementary Materials/Supplementary Folder 1.zip!/Oidma1_AssemblyScaffolds_Repeatmasked/knownclusterblast/cluster21/jgi.p_Oidma1_165267_mibig_hits.html]

| MIBiG Protein | Description | MIBiG Cluster | MiBiG Product | % ID | % Coverage | BLAST Score | E-value |
| --- | --- | --- | --- | --- | --- | --- | --- |
| EFG04600.1 | Staurosporine\_biosynthesis\_methyltransferase\_StaMA | BGC0000826 | Staurosporine\_biosynthetic\_gene\_cluster | 25 | 36.8778280543 | 66 | 9.6e-11 |
| ABC02795.1 | D-glucose\_O-methyltransferase | BGC0000809 | AT2433\_biosynthetic\_gene\_cluster | 29 | 22.850678733 | 59 | 9e-09 |
| AFZ08007.1 | Methyltransferase\_type\_11 | BGC0000471 | Viridisamide\_A\_biosynthetic\_gene\_cluster | 28 | 27.8280542986 | 53 | 8.4e-07 |
| CCH92966.1 | putative\_Sterol\_24-C-methyltransferase | BGC0000483 | Aeruginosamide\_biosynthetic\_gene\_cluster | 28 | 27.149321267 | 52 | 1.1e-06 |
| CAN93350.1 | polyketide\_synthase | BGC0000179 | Etnangien\_biosynthetic\_gene\_cluster | 28 | 43.4389140271 | 51 | 2.4e-06 |
